# Predictors of Survival Among Preterm Neonates Admitted to Felege Hiwot Comprehensive Specialized Hospital, Northwest Ethiopia

**DOI:** 10.3389/fped.2022.800300

**Published:** 2022-03-10

**Authors:** Demeke Mesfin Belay, Workie Zemene Worku, Amare Wondim, Habtamu Shimels Hailemeskel, Wubet Alebachew Bayih

**Affiliations:** ^1^Department of Pediatrics and Child Health Nursing, College of Health Sciences, Debre Tabor University, Debre Tabor, Ethiopia; ^2^Department of Community Health Nursing, College of Medicine and Health Sciences, University of Gondar, Gondar, Ethiopia; ^3^Department of Pediatrics and Child Health Nursing, College of Medicine and Health Sciences, University of Gondar, Gondar, Ethiopia; ^4^Department of Maternity and Neonatal Health Nursing, College of Health Sciences, Debre Tabor University, Debre Tabor, Ethiopia

**Keywords:** Ethiopia, predictors, survival, preterm, neonates

## Abstract

**Background:**

Pre-maturity is the primary cause of neonatal mortality in the world. Although prematurity was the leading cause of neonatal mortality, the survival rate and its predictors may be varied from setting to setting and time to time due to different reasons. Therefore, this study aimed to assess the survival probability and predictors of mortality among preterm neonates at Felege Hiwot comprehensive specialized hospital.

**Methods:**

This is a retrospective follow-up study that included 542 randomly selected preterm neonates admitted at Felege Hiwot comprehensive specialized hospital from the period of 2016-2020. Semi-parametric and parametric survival models were fitted to identify the survival probability of preterm neonates and its association with different predictors. The best fit model was selected using Akaike's information criteria, Bayesian information criteria and likelihood ratio criteria.

**Results:**

The cumulative incidence and incidence rate of mortality among preterm neonates were 31 per 100 live births and 3.5 per 100 neonate days, respectively. From the adjusted cox-proportional-hazard model, predictors with higher preterm mortality risk include the presence of neonatal respiratory distress syndrome [AHR = 2.55, 95% CI: 1.23; 3.74], perinatal asphyxia [AHR = 4.26, 95% CI: 1.35; 6.79] and jaundice [AHR = 3.25, 95% CI: 2.14, 7.24]. However, admission weight of 1,500–2,499 g (AHR = 0.23, 95% CI: 0.11, 0.56) and ≥2,500 g (AHR = 0.12, 95% CI: 0.02; 0.32), early breastfeeding [AHR = 0.44, 95% CI: 0.36; 0.48] and kangaroo mother care [AHR = 0.11, 95% CI: 0.03; 0.15] were protective factors of preterm mortality.

**Conclusion:**

The cumulative incidence of mortality among preterm neonates was consistent with the national incidence of preterm mortality. Factors such as respiratory distress syndrome, perinatal asphyxia, breastfeeding, kangaroo mother care, admission weight, and jaundice are significant predictors of survival. Therefore, considerable attention such as intensive phototherapy, optimal calorie feeding, oxygenation, and good thermal care should be given for admitted preterm neonates.

## Background

The World Health Organization (WHO) defines preterm birth as a baby born before 37 completed weeks of gestation (1). It can be categorized into extremely preterm (<28 weeks of gestation), very preterm (28 to <32 weeks of gestation), and moderate preterm (32 to <37 weeks of gestation) (2, 3). Preterm birth is the primary cause of neonatal mortality and the second cause of under-five mortality in the world (4). Every year, an average of one million neonates and under-five children die of its complications (5). The survival rate in high and low-income countries is considerably varied with 9 and only 1 survivor out of the 10 preterm babies, respectively (2). The mortality among Africans showed twelve times higher compared to Europeans. In Sub-Saharan Africa, preterm birth is the second main cause of under-five mortality, accounting for 12.1% of deaths, and more than 50% of neonatal death in the East Africa is attributable to preterm birth (6). Furthermore, the survivor's are usually at a higher risk of morbidity and life-long physical, neurological, visual, learning, and hearing disabilities (7–11).

Among the neonates, the protective and risk factors associated with mortality including intra-natal related factors, post-natal related factors, maternal obstetrics related factors, neonatal related factors, and maternal socio-demographic related factors have been identified previously (8, 12, 13).

The majority of premature babies can be saved by feasible and cost-effective care using the continuity of midwifery-led care that could reduce the risk of prematurity by around 24 % (2). In doing so, Ethiopia has been incorporated the new WHO recommendations for improving preterm birth outcomes in the clinical standard (14). Moreover, the country has proposed a national strategy for newborn and child survival from 2015 to 2020 to end all preventable newborn and child deaths by 2035 (15). On the other hand, the Sustainable Development Goal three (SDG-3) offers a great weight to decrease neonatal mortality with a target of 12 neonatal deaths per 1,000 live births by 2030 (16). Despite these and many other efforts, the prevalence of neonatal mortality among preterm neonates is high. Different studies have been done on preterm neonates in Ethiopia; however, the majority of them used <4 year's data (17–20), and all of these studies did not investigate the independent predictors (time of breastfeeding initiated, gestational age and admission/birth weight) of preterm survival (17–23). Furthermore, some studies employed a cross-sectional type of study design, usually unable to make a causal inference (17, 20). Therefore, the goal of this study was to identify predictors (time of breastfeeding initiated, admission/birth weight, and gestational age) of survival among preterm neonates using 5 years retrospective follow-up study design.

## Methods

### Study Design and Setting

A retrospective follow-up study was carried out on preterm neonates admitted to the neonatal intensive-care unit at Felege Hiwot comprehensive specialized hospital. The hospital is located in Bahir Dar city, 552 km away from Addis Ababa, the capital city of Ethiopia. It is a public hospital serving a total of about 5 million populations. Most of the mothers who delivered in the hospital (>85%) have at least four antenatal care follow-up (24). The hospital has an annual delivery rate of about 6,000. Though the usual deliveries are from the hospital, it also serves some deliveries referred from the district, general, and referral hospitals in Amhara region. Nearly 3.4% of the total deliveries in the hospital were reported with neonatal mortality. Neonatal admissions in 2020 were 4,584, of whom the majorities (about 61%) were preterm neonates, including both the out born and inborn neonates. Preterm admission criteria were gestational age <34 weeks at birth or any preterm neonates with complications (having at least one newborn danger sign). About three-fourths of the overall neonatal admissions are from hospital deliveries (inborn). Out born from the different parts of Amhara region including the city are also admitted to the NICU. Out born neonates are transported from lower-level hospitals to the hospital NICU by ambulance with neonatal care providers. The neonatal intensive-care unit has five different room's namely preterm neonate rooms, term room, kangaroo mother care room, septic neonate room, and mother side room. In the NICU, there are different equipment including oxygen tubing, incubators, photo-therapy machine, CPAP machines, heaters and radiant warmers. Furthermore, there are therapeutic interventions such as intubation, vasopressin, IV antibiotics, gavage feeding, and parenteral nutrition. However, preterm services like surfactant administration and plastic wrapping aren't available in the study setting (24).

### Source and Study Population

All preterm neonates admitted to neonatal intensive care unit at Felege Hiwot comprehensive specialized hospital were considered as a source population whereas all preterm neonates who were admitted to NICU at Felege Hiwot comprehensive specialized hospital from a period of January 1, 2016, to December 30, 2020, were the study population. The gestational age was determined by first-trimester ultrasound results for mothers with available reports or Ballard score for those mothers without the reports.

### Eligibility Criteria

Preterm neonates with incomplete medical records (i.e., if the outcome, date of admission, date of death/censored, and the gestational age are not recorded) and gross congenital malformation were excluded.

### Sample Size Determination

A total sample size of 542 preterm neonates was obtained using STATA version 14 statistical software based on the cox-proportional hazard regression, taking a Crude Hazard Ratio (CHR) of different predictors such as home delivery (CHR = 2.14), hyaline membrane disease (CHR = 4.38), KMC (CHR = 0.41), crying immediately after birth (CHR = 0.25) and hypoglycemia (CHR = 2.1) as effect size (20), 5% marginal error, a standard error of 0.5, study power of 80% and a reasonable estimate for the proportion of preterm mortality from the prior study was 28.8% (25). Meanwhile, to check the adequacy of the sample size used, a *post-hoc* power analysis was computed and the sample size was adequate to represent the study population with *post-hoc* power value of 90%.

### Sampling Technique and Procedures

**Figure 1:**
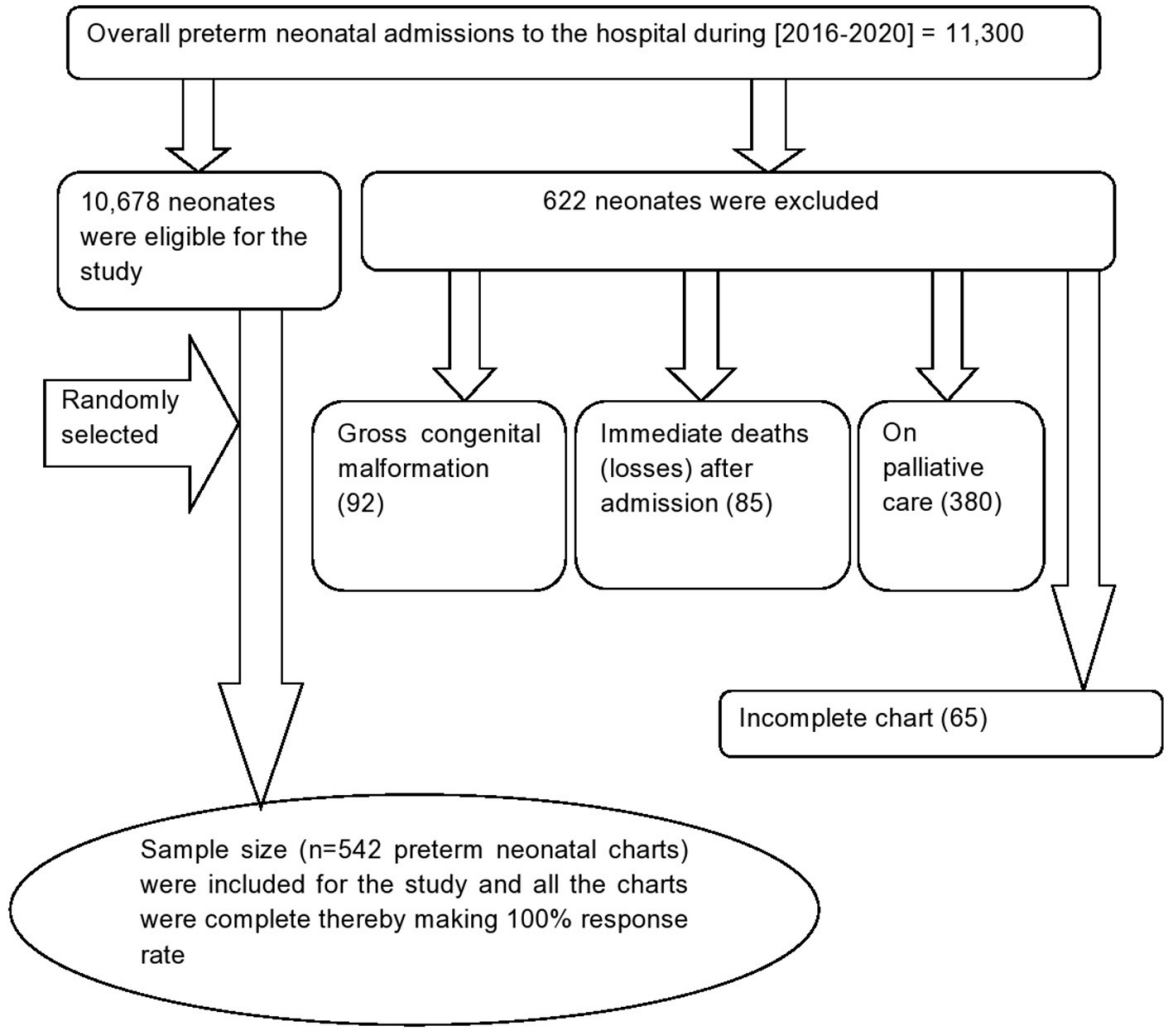
Flow diagram to show the overall admission of preterm birth Felege Hiwot comprehensive specialized hospital, Northwest Ethiopia, 2016-2020.

Medical Record Numbers (MRN) of the eligible preterm neonates charts were first coded by the Health Information Management system (HMIS) focal persons. Then, the eligible preterm neonate's were included by a computer-generated random selection of the coded neonatal medical charts (Figure 1).

### Data Collection Procedures and Quality Assurance

The data were collected by four trained clinical nurses under the supervision of a public health officer. Abstractors used a standardized case report form to retrieve the following information from medical charts: maternal socio-demographics, antenatal history, delivery history, neonatal complications, and treatments. Assessment of inter-rater reliability on 43 items on the case report form revealed a Cronbach alpha of 0.81. Furthermore, the case report form was validated through pre-testing on 28 preterm neonatal charts (5% of the sample size) 2 weeks before the actual data collection time at the same hospital.

### Variables in the Study

#### Event

Eligible preterm neonate who died during the follow-up time (from date of admission until discharge).

#### Censored

Preterm neonates who were referred to other health institutions or discharged with parental refusal or alive during discharge were considered as censored.

#### The Time Origin

Admission of preterm neonates to NICU at Felege Hiwot comprehensive specialized hospital.

#### Follow-Up Time

From the time of admission until either an event or censorship occurs.

#### Time to Event

The time elapsing from the date of admission at NICU of the study hospital to the occurrence of death before discharge, which can be calculated by subtracting the date of death from the date of admission.

**Obstetrics complications** are considered if the mother had one or more of the following problems such as Premature Rupture of Membranes (PROM), corioamnioitis, eclampsia/pre-eclampsia and polyhaydraminos (15, 26).

**Neonatal complications** are considered if the neonate had one or more of the following problems such as Perinatal Asphyxia (PNA), Necrotizing Enterocolitis (NEC), jaundice, hypothermia, sepsis, hypoglycemia, and Respiratory Distress Syndrome/Hyaline Membrane Disease (RDS/HMD) (7, 11, 15).

Other variables such as Small for Gestational Age (SGA), Appropriate for Gestational Age (AGA), hypothermia, hypoglycemia, jaundice, antenatal visit, polyhaydraminous, PNA, NEC, PROM, chorioamnionitis, RDS/HMD, KMC, and use of antenatal steroids if the diagnosis was made at the admission of neonates to NICU.

Pre-maturity is defined as a baby born alive before 37 completed weeks of gestation (1).

### Data Management and Analysis

The collected data were coded and double entered into Epi-info statistical software version 7.2.01 and exported to STATA version 14 statistical software for analysis. Since the proportion of missing data was not more than 5% for all variables, we used complete case-analysis. Descriptive statistics were carried out and summarized using means, medians, and proportions. The cumulative incidence of NICU mortality was determined to be the number of neonatal deaths per 100 live neonatal births while the incidence rate of preterm mortality among neonatal admissions to NICU was determined using Neonate-Day (ND) follow-up as a denominator for the entire cohort and for the group classified based on socio-demographic and other variables. The Kaplan-Meier curve was used for the analysis of the probabilities of preterm mortality. The log-rank test was used to compare survival curves between groups of explanatory variables. A life table was used to estimate the probability of survival at different time intervals in the follow-up time. Variables with a *p*-value <0.2 in bi-variable cox-proportional hazard models were entered into multi-variable cox-proportional hazard models to identify predictors of survival among preterm neonates. A 95% confidence interval of the hazard ratio was computed and variables with a *p*-value ≤ 0.05 in the multi-variable cox-proportional hazard model were considered as significant predictors of the incidence of mortality among preterm neonates.

### Model Comparison and Goodness of Fit Test

The cox-proportional hazard model and other parametric survival analysis models (Gompertz Distribution, Wei-bull Distribution, and Exponential Distribution) were fitted by considering the baseline hazard distribution assumptions into account. Hence, the final fitted model, the cox-proportional hazard model was selected based on Akaike's information criteria, Bayesian information criteria, and likelihood ratio. The overall model fitness was assessed by using a cox-Snell residual test.

### Ethical Approval and Consent to Participate

Ethical approvals were obtained from the University of Gondar, College of Medicine and Health Science, Institutional Health Research Ethics Review Committee (IHRERC). Informed consent was waived because the study was conducted using secondary data. To further secure the ethical perspective of the study, a permission letter was issued from Felege Hiwot comprehensive specialized hospital administration. Moreover, results were reported compositely in groups to secure confidentiality.

## Results

### Maternal Socio-Demographic and Obstetrics Related Characteristics

The mean maternal age was 26.64 (±5.24) years. Three-fifth (60.1%) of the mothers were urban residents, and a nearly equal number of pregnancies, 331 (61.1%) resulted in single tone birth. Five hundred ten (94.10%) mothers had antenatal care follow-up. One hundred fifty-five (28.60%) mothers delivered outside Felege Hiwot comprehensive specialized hospital (Table 1).

**Table 1:** Maternal socio-demographic and obstetrics related characteristics at FHCSH, during 2016-2020, (*n* = 542).

**Covariate**	**Survival status (*****n*** **=** **542)**	
	**Censored**	**Event**
**Maternal age**		
<20 year	242	106
20–34 years	117	56
>=35	15	6
**Maternal residency**		
Rural	144	72
Urban	230	96
**Parity**		
1	181	75
2–4	139	66
>=5	54	27
**Premature rupture of membrane**		
No	281	145
Yes	93	23
**Pre-eclampsia/Eclampsia**		
No	325	140
Yes	49	28
**Pregnancy induced hypertension**		
No	315	140
Yes	59	28
**Maternal HIV/AIDS**		
No	366	166
Yes	8	2
**Mode of delivery**		
SVD	303	135
C/S	71	33
**Current pregnancy**		
Single	225	106
Multiple	149	62
**Antenatal corticosteroid given (*****n*** **=** **138)**		
No	70	30
Yes	28	10
**Chorioamnionitis**		
No	357	162
Yes	17	6
**Polyhydraminous**		
No	365	158
Yes	9	10
Overall mortality	374	168

### Socio-Demographic and Clinical Characteristics of Preterm Neonates

Out of 11,300 neonatal admissions to NICU in the hospital during the 5 years period [2016–2020], 622 preterm neonates were excluded for different reasons as illustrated in the flow diagram (Figure 1). About three-fifth [333 (61.44%)] of the preterm neonates were females. The mean gestational age at birth was 32.9 (95% CI = 32.67, 33.16) weeks, and about one-eighth (12.5%) of the preterm neonates were small for gestational age. More than half (306, 56.4%) of the preterm neonates have not initiated breastfeeding within the first hour of birth. More than two-thirds (374, 69%) of preterm neonates did not get KMC services. Besides, more than three quarters (420 77.49%) of the neonates have hypothermia (Table 2).

**Table 2:** Socio-demographic and clinical characteristics of preterm neonates at FHCSH, 2016-2020, (*n* = 542).

**Gestational age (weeks)**	***N***
<32 weeks	118
>=32 weeks	424
**Admission weight (g)**	
1,000–1,499 g	155
1,500–2,499 g	340
>=2,500 g	47
**1**^**st**^ **min Apgar score**	
0–3	22
4–6	177
7–10	343
**5**^**th**^ **min Apgar score**	
0–3	11
4–6	174
7–10	355
**RDS**	
No	328
Yes	214
**Weight for gestational age**	
AGA	474
SGA	68
**Sepsis**	
No	117
Yes	425
**PNA**	
No	505
Yes	37
**Jaundice**	
No	343
Yes	199
**Hypoglycemia**	
No	477
Yes	65
**NEC**	
No	445
Yes	97
**KMC**	
No	374
Yes	168
**Time of breast feeding initiated**	
Within 1 h	236
>1 h	306

### Incidence of Preterm Mortality

**Figure 2:**
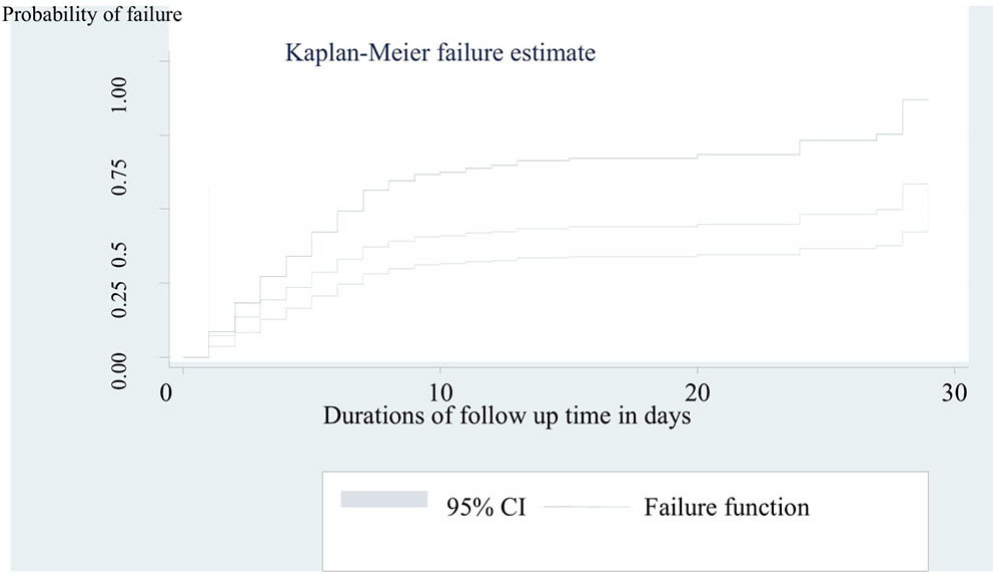
Kaplan-Meier curve to estimate failure of preterm neonates admitted to NICU at FHCSH from 2016 to 2020 (*n* = 542).

The cumulative incidence of neonatal mortality among preterm neonates was 31 per 100 live births. In the mean follow-up period of 40 days within the range of 32 to 48 follow-up months, the incidence rate of preterm mortality was 3.5 per 100 neonate days (95% CI = 1.9, 5.1). The median survival time was 30 days (95% CI = 6, 37) in a total of 4,820 neonates-days observation (Figure 2). In this cohort, the incidence rate of mortality among preterm neonates who were not under KMC service was 5.8 per 100 neonate days (95% CI = 3.8, 7.8). Furthermore, the incidence rate of mortality among preterm neonates who had PNA was 10 per 100 neonates days (95% CI = 7.5, 12.5). The incidence rate of preterm mortality was also found to be 6.2 per 100 neonate days (95% CI = 4.2, 8.2) among preterm neonates who initiated breastfeeding after 1 h of birth.

### Comparison of Survival Experience

**Figure 3:**
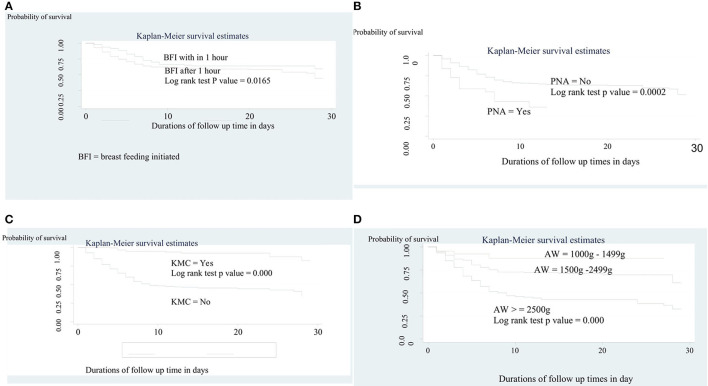
**(A)** The Kaplan-Meier survival curves by time of initiation of breast feeding among preterm neonates admitted to NICU at FHCSH, 2016-2020 (*n* = 542). **(B)** The Kaplan-Meier survival curves by KMC service among preterm neonates admitted to NICU at FHCSH, 2016-2020 (*n* = 542). **(C)** The Kaplan-Meier survival curves by PNA among preterm neonates admitted to NICU at FHCSH, during 2016-2020 (*n* = 542). **(D)** The Kaplan-Meier survival curves by admission weight among preterm neonates admitted to NICU at FHCSH, 2016-2020 (*n* = 542).

The overall survival across various groups was compared using a log-rank test. We found significant differences in the overall survival across various groups including PNA, time of breastfeeding initiated, admission weight, and KMC (Figures 3A–D). Accordingly, there was a clear statistically significant mortality difference between preterm neonates who initiate breastfeeding within 1 hour of birth [21 days (95% CI = 18, 23)] and their counterpart [18 days (95% CI = 17, 20)] (*p* = 0.0165). On the other hand, the mean survival time of preterm neonates who had not received KMC was significantly higher than 27 days (95 % CI = 25, 28) that of the received [15 days (95% CI = 14, 17)] (*p* = 0.000). A similar result has been recorded among preterm neonates who had not PNA (19 days 95% CI = 18, 21), compared to those who had PNA [7 days (95% CI = 5, 9)] (*p* = 0.0002). Moreover, the mean survival time among those who had admission weight of 1,000–1,499 g was significantly lower (14 days, 95% CI = 12, 17) than those with an admission weight of 1,500–2,499 g (21 days, 95% CI = 20, 23) and ≥2,500 g (24 days, 95% CI = 22, 27) (*p* = 0.000).

### Assessing the Proportional Hazard Assumption

The global test of proportional-hazards assumption based on the Schoenfeld residuals revealed that all of the co-variates and full model satisfies the cox-proportional hazard assumption at *p* = 0.2425.

### Model Selection

To identify predictors of survival among preterm neonates, semi-parametric and parametric proportional hazard models were fitted. The most parsimonious model was chosen using AIC, BIC, and LR, and the cox-proportional hazard model (AIC = 277.7724, BIC = 369.7376, log-likelihood = 114.8862) was found to be more efficient than other parametric models (Table 3). Interpretations and conclusions were thus based on the cox-proportional hazard model.

**Table 3:** Summary of Model comparison between semi-sox proportional hazard models and parametric Cox- Regression models using AIC, BIC and log likelihood.

**Comparison methods**	**Models**			
	**Cox PH model**	**Exponential**	**Weibull**	**Gompertz**
Log likelihood	−114.8862	−167.57514	−115.9195	−408.4529
AIC	277.7724	381.1515	279.8319	860.9258
BIC	369.7376	469.2848	371.7971	945.2272

### The Goodness of Fit Test

**Figure 4:**
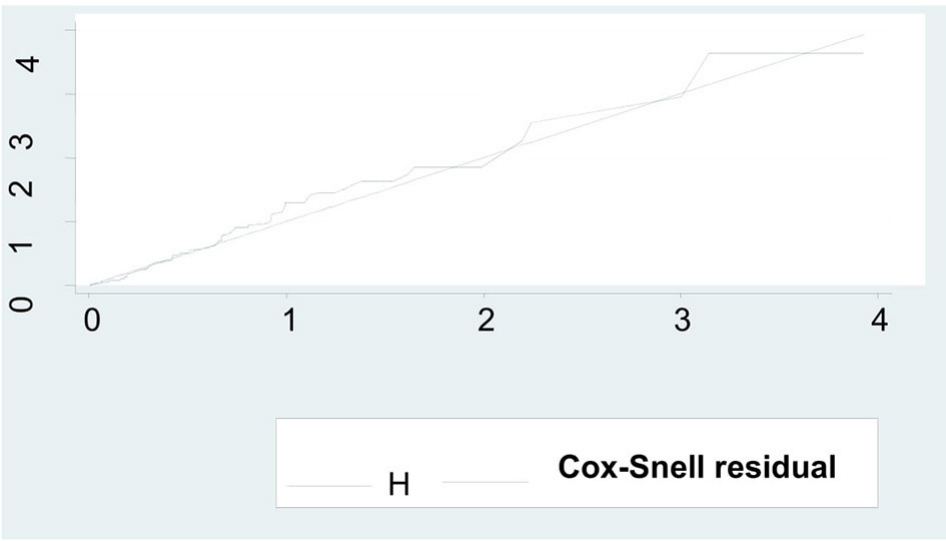
Model fitness by using Cox-Snell residual test among preterm neonates admitted to NICU at FHCSH, during 2016-2020 (*n* = 542).

It can be seen that the plot of the Nelson-Allen cumulative hazard function against Cox-Snell residuals is closest to 45^0^ straight lines through the origin for the cox-proportional hazard model when compared to the parametric survival model. This suggested that the cox-proportional hazard model provided the best fit for our data set (Figure 4).

### Multi-Variable Analysis of Cox-Proportional Hazard Model

From the bi-variable cox-regression analysis, PNA, jaundice, KMC, gestational age, RDS, admission weight of 2,500 g and above, initiation of breastfeeding within 1 h of birth, and first minute Apgar score were significant predictors of preterm mortality. However, after adjusting for possible confounders in multi-variable cox-regression analysis, PNA, jaundice, KMC, RDS, initiation of breastfeeding within 1 h of birth, and admission weight of 2,500 g and above were independently and significantly associated with the incidence rate of preterm mortality.

The hazard of mortality among preterm neonates who had PNA was 4.3 times higher as compared with those who had not PNA (AHR = 4.26, 95% CI: 1.35; 6.79). In addition, the risk of mortality among preterm neonates diagnosed with RDS was 2.6 times higher as compared to those who had not RDS (AHR = 2.55, 95% CI:1.23; 3.74). The risk of mortality among preterm neonates who had jaundice was 3.3 times higher than their counterparts (AHR = 3.25, 95% CI: 2.14, 7.24).

Preterm neonates who had admission weight of 1,500–2,499 g (AHR = 0.26, 95% CI: 0.11, 0.56) and ≥2,500 g (AHR = 0.12, 95% CI: 0.02; 0.32) had 74 and 88% less hazard of mortality as compared with those who had admission weight of 1,000–1,499 g.

Preterm neonates who initiated breastfeeding within 1 h of birth had 56% less hazard of mortality as compared with their counterparts (AHR = 0.44, 95% CI: 0.36; 0.48). Providing KMC for preterm neonates reduce the risk of mortality by 89% as compared with their counterparts (AHR = 0.11, 95% CI: 0.03; 0.15) (Table 4).

**Table 4:** Adjusting for sex, bi-variable and multivariable Cox regression analysis of predictors of incidence of mortality among preterm neonates admitted to NICU at FHCSH from 2016 to 2020 (*n* = 542).

**Predictors**	**Survival status**		**CHR (95% CI)**	**AHR (95% CI)**	***P*-value**
	**Censored**	**event**			
**Perinatal asphyxia**					
No	358	147	1	1	
Yes	17	20	3.44 (2.51; 4.52)	4.26 (1.35; 6.79)	0.023
**Jaundice**					
No	234	109	1	1	
Yes	141	58	2.41 (1.52, 6.62)	3.25 (2.14, 7.24)	0.014
**KMC**					
No	219	155	1	1	
Yes	156	12	0.16 (0.04, 0.19)	0.12 (0 0.03; 0.15)	0.011
**Time of breast feeding**					
Within 1 h	182	54	0.68 (0.48; 0.92)	0.44 (0.36; 0.48)	0.015
>1 h	193	114	1	1	
**Admission weight**					
1,000–1,499 g	66	89	1	1	
1,500–2,499 g	267	73	0.31 (0.24; 0.57)	0.26 (0.11, 0.56)	0.007
>=2,500 g	42	5	0.25 (0.17; 0 0.48)	0.12 (0.02; 0.32)	0.029
**Gestational age**					
<32 weeks	54	64	0.41(0.23; 0.68)	1.02 (0.51; 1.79)	0.089
> = 32 weeks	321	103	1	1	
**RDS**					
No	272	56	1	1	
Yes	103	111	3.12 (2.13; 4.56)	2.55 (1.23; 3.74)	0.002
**1**^**st**^ **min Apgar**					
0–3	7	15	0.11 (0.32, 0.60)	0.34 (0.64, 1.29)	0.065
4–6	111	66	0.87 (0.15; 0.94)	0.23 (0.89; 8.77)	0.23
7–10	257	86	1	1	
**Sex**					
Male	248	85	1	1	
Female	173	36	2.54 (0.43, 3.87)	4.3 (0.12, 5.77)	0.34

When gestational age below 32 weeks and admission weight (1,000–1,499 g) were entered together in the final model (were controlled each other) as presented in Table 4, gestational age wasn't significant. This may be due to co-linearity between these variables (standard error = 2.13). Thus, on separate entry to the final model, while adjusting for sex; gestational age below 32 weeks became significant. Adjusting for sex, preterm neonates whose gestational age below 32 weeks had 5.8 times more hazard of mortality (AHR = 5.78, 95% CI, 4.03; 8.23) as compared to those whose gestational age was above 32 weeks when admission weight (1,000–1,499 g) was not entered to the model (Table 5).

**Table 5:** Significance of gestational age (<32 weeks) in the multivariable cox proportional hazard model when the model is adjusted for sex and admission weight (1,000–1,499 g) was not entered to the model.

**Factors**	**Survival status**		**CHR (95% CI)**	**AHR (95% CI)**	***P*-value**
	**Censored**	**event**			
**Perinatal asphyxia**					
No	358	147	1	1	
Yes	17	20	3.44 (2.51; 4.52)	2.56 (1.50; 4.37)	0.001
**Jaundice**					
No	234	109	1	1	
Yes	141	58	2.41 (1.52, 6.62)	3.17 (1.21, 6.52)	0.034
**KMC**					
No	219	155	1	1	
Yes	156	12	0.16 (0.04, 0.19)	0.11 (0 0.02; 0.24)	0.023
**Time of breast feeding**					
Within 1 h	182	54	0.68 (0.48; 0.92)	0.37 (0.28; 0.67)	0.003
>1 h	193	114	1	1	
**Admission weight (*****n*** **=** **387)**					
1,500–2,499	267	73	4.57 (2.31; 7.83)	7.23 (3.23, 9.88)	0.000
>=2,500	42	5	1	1	
**Gestational age**					
<32 weeks	54	64	0.41 (0.23; 0.68)	5.78 (4.03; 8.23)	0.026
>=32 weeks	321	103	1	1	
**RDS**					
No	272	56	1	1	
Yes	103	111	3.12 (2.13; 4.56)	4.37 (2.36; 5.61)	0.012
**1**^**st**^ **min Apgar**					
0–3	7	15	0.11 (0.32, 0.60)	0.25 (0.14, 1.24)	0.32
4–6	111	66	0.87 (0.15; 0.94)	0.34 (0.15; 7.55)	0.17
7–10	257	86	1	1	
**Sex**					
Male	248	85	1	1	
Female	173	36	2.54 (0.43, 3.87)	1.92 (0.26, 6.78)	0.25

Furthermore, adjusting for sex and leaving gestational age from the model, preterm neonates whose admission weight (1,000–1,499 g) had 4.7 times more hazard of mortality (AHR = 4.65, 95% CI = 2.27, 6.70) as compared to those whose admission weight was ≥2,500 g (Table 6).

**Table 6:** Significance of admission weight (1,000–1,499 g) in the multivariable cox proportional hazard model when the model was adjusted for sex and gestational age (<32 weeks) was not entered to the model.

**Covariate**	**Survival status**		**CHR (95% CI)**	**AHR (95% CI)**	***P*-value**
	**Censored**	**event**			
**Perinatal asphyxia**					
No	358	147	1	1	
Yes	17	20	3.44 (2.51; 4.52)	2.56 (1.67; 6.88)	0.021
**Jaundice**					
No	234	109	1	1	
Yes	141	58	2.41 (1.52, 6.62)	4.17 (2.35, 7.81)	0.041
**KMC**					
No	219	155	1	1	
Yes	156	12	0.16 (0.04, 0.19)	0.21 (0 0.07; 0.68)	0.013
**Time of breast feeding**					
Within 1 h	182	54	0.68 (0.48; 0.92)	0.47 (0.19; 0.58)	0.004
>1 h	193	114	1	1	
**Admission weight (g) (*****n*** **=** **542)**					
1,000–1,499	35	120	1.33 (2.31; 6.81)	4.65 (2.27, 6.70)	0.016
1,500–2,499	267	73	3.45 (2.31; 6.81)	3.11 (2.18, 9.21)	0.039
>=2,500	42	5	1	1	
**RDS**					
No	272	56	1	1	
Yes	103	111	3.12 (2.13; 4.56)	2.41 (1.88; 4.33)	0.027
**1**^**st**^ **min APGAR score**					
0–3	7	15	0.11 (0.32, 0.60)	0.75 (0.42, 1.99)	0.54
4–6	111	66	0.87 (0.15; 0.94)	0.88 (0.24; 7.55)	0.84
7–10	257	86	1	1	
**Sex**					
Male	248	85	1	1	
Female	173	36	2.54 (0.43, 3.87)	1.75 (0.14, 3.62)	0.65

## Discussion

This retrospective follow-up study aimed to assess the incidence rate of mortality and its predictors among preterm neonates admitted to NICU at Felege Hiwot comprehensive specialized hospital. In this study, the incidence rate of preterm mortality was 3.5 per 100 neonate days. Moreover, the cumulative incidence of preterm neonatal mortality was 30.81% which is consistent with the study conducted in Gondar, Addis Ababa, Nigeria, East Africa, and Iran (12, 14, 20, 27, 28). However, this finding was higher than the study conducted in Nairobi hospital in Kenya, Malawi, and Mulago National referral hospital in central Uganda (29–31). The possible reasons could be difference in the study participants between the studies. Thus, the study participants in our study were both inborn and out born preterm neonates which might have increased the time-lapse for starting appropriate neonatal care. However, the study in Malawi (29) included out born babies when they were <48 h. In addition, the majority of the study participants in Mulago National referral hospital in central Uganda were inborn preterm neonates (30). On the other hand, the disparity in the study period contributed to the variation of the findings between the studies.

But, the finding in our study was lower than the study conducted in Felege Hiwot comprehensive specialized hospital, Jimma, Eritrea, Bangladesh, and India (18, 19, 32–34). This discrepancy may be due to differences in study populations which include preterm neonates who had gestational age 28 weeks and above with different categories of birth weight in this study but the study populations were preterm neonates who had gestational age 26 weeks and above in Jimma (19) and very low birth weight preterm neonates in India (32). Moreover, the study design in Felege Hiwot comprehensive specialized hospital and Eritrea (18, 33) was cross-sectional in sharp contrast to the retrospective follow-up in our study. The duration of follow-up time was only seven days in Bangladesh (34) and forty in our study.

On the other hand, the median survival time in this study was 30 days (95% CI = 6, 37) which is consistent with the study conducted in Black lion specialized hospital (28 days) (35), University of Gondar comprehensive specialized hospital (21 days) (21), and Mizan Tepi teaching hospital (15 days) (17). The similarity in the quality of service provided between the hospitals may contribute to the consistency. Therefore, to enhance the survival rate of preterm birth different specialty neonatal care has to be provided during the first month of the neonatal period.

From the adjusted cox-regression analysis, the hazard of mortality among preterm neonates who had perinatal asphyxia was 4.3 times higher than those who had no perinatal asphyxia. This finding was congruent with the study conducted in Gondar, Jimma and Ethiopia suggested that perinatal asphyxia was the commonest cause of mortality among premature neonates (19, 20, 36). This is related to intra-partum hypoxia, which contributed to difficulties in the transition to the extra-uterine environment. Hence, for preterm neonates who had prenatal asphyxia, lifesaving essential and extra essential care like neonatal resuscitation and safe oxygen use should be encouraged. Besides, advanced neonatal care such as surfactant replacement has to be provided for preterm neonates with RDS secondary to PNA (37). However, it is not routine practice in our study setting. But, an Indian study has shown that perinatal asphyxia was not a significant predictor of neonatal mortality among preterm neonates. This could be due to differences in study populations because our study was entirely on preterm neonates whereas the study in Indian include only very low birth weight neonates (32).

Preterm neonates who had admission weight of 1,500–2,499 g and ≥2,500 g had 74 and 88% less hazard of mortality as compared to those who had admission weight of 1,000–1,499 g, respectively. This result is supported by the study conducted in Gondar, Addis Ababa, Eritrea, East Africa, Iran, China, western Nepal, and the Eastern Mediterranean region (14, 27, 28, 35, 38–41). This congruence may be due to the fact that low birth weight preterm neonates have low levels of brown fat that predisposes them to hypoglycemia and hypothermia thereby increasing their risk of mortality. Physiologically, these neonates have immature organ development (especially the lungs), have poor feeding skills, metabolic deregulation, and are at a higher risk of infections, all of which could increase susceptibility for different preterm birth complications and death. In the study setting, neonatal complications such as hypothermia and hypoglycemia are high. However, KMC was poorly practiced in the study setting. Therefore, WHO thermal care for preterm neonates such as KMC, radiant warmers, and/or incubators are recommended (42) to prevent hypothermia and hypoglycemia-related mortality of preterm neonates.

Preterm neonates who had jaundice were at a higher hazard of mortality than those who had not jaundiced. This may be due to adverse outcomes due to either neurotoxicity or over-treatment of photo-therapy or exchange transfusion (43). Moreover, jaundice considered for this study was pathological type as operationalized in the methods section and hence, the jaundiced preterm neonates were more likely to develop kernicterus because the hypoxia from respiratory distress and perinatal asphyxia accelerates communication of the preterm neonate's un-conjugated hyper-bilirubin with their neuronal tissues thereby increases the likelihood of acute bilirubin encephalopathy (kernicterus) (7, 11). Besides, some of the pathologically jaundiced preterm neonates admitted to the hospital were attributed to ABO and/Rh incompatibility with severe anemia as ABO/Rh sensation was a common phenomenon in the study setting during the study period (24). As a result, regular and timely monitoring of bilirubin level is helpful to prevent treatment-related complications of jaundice (neurotoxicity or over-treatment of photo-therapy or exchange transfusion) and to identify the type of treatment provided for preterm neonates with jaundice. In addition, a screening test for bilirubin level has to be performed for preterm neonates who had PNA and/or RDS.

The hazard of mortality among preterm neonates who had RDS was 2.6 times higher as compared with their counterparts. This finding was similar to the study conducted in Gondar, Jimma, Ethiopia, Iran, and India (19, 20, 28, 35, 44, 45). Neonatal RDS is often accompanied by life-threatening complications such as pneumothorax, pulmonary hypertension, Cor-Pulmonary and metabolic deregulations which in turn increase the possibility of preterm mortality (46–48). Hence, to reduce the risk of RDS, antenatal corticosteroids are WHO-recommended practice for women at risk for preterm labor and/or in preterm labor (13).

In our study, receiving KMC service and early breastfeeding were protective factors of preterm mortality. Comparison of mortality risk regarding KMC and early breastfeeding services was made among preterm neonates who were relatively stable (denominator). Despite eligibility for KMC and early breastfeeding, some neonates weren't given these cares because of probably different constraints in the study setting and even from the parental side, which in turn necessitates another investigation (24). The hazard of death among preterm neonates who received KMC was decreased by 89%. This may be due to the fact that KMC service facilitates skin-to-skin contact, optimal breastfeeding, and prevention of apnea and colonization of the neonate's skin with mutualistic maternal skin microbial flora. These clinical significances of KMC help prevent the neonates from being hypothermia, hypoglycemic, distressed, and developing infection thereby increasing their survival (33, 35, 45, 49, 50). Therefore, preterm infants whose birth weight is <2,000 g and have relatively stable vital signs should always receive KMC service to optimize their survival.

The hazard of death among preterm neonates who initiated breastfeeding within 1 h of birth was 56% less as compared to those who initiated later. This finding was supported by the study conducted in Gondar and India (20, 32). This may be due to the fact that early initiation of breastfeeding decreases the risk of hypoglycemia, infection, and hypothermia (49, 51–54). For this reason, the relatively stable preterm neonate should be initiated breastfeeding as early as possible after gut priming is once tolerated (7, 11).

## Strength and Limitation of the Study

This study included study populations from a tertiary center, which handled large number of high-risk preterm neonates. Since the data were accessed from secondary source; some important predictors such as socio-economic and nutritional statuses were missed which are significant predictors of mortality among preterm neonates. Furthermore, the study area covers only Felege Hiwot comprehensive specialized hospital that limit's representations to other hospitals found in Amhara region and Ethiopia.

## Conclusion

The cumulative incidence of mortality among preterm neonates was consistent with the national incidence of preterm neonatal mortality. Factors such as respiratory distress syndrome, perinatal asphyxia, breastfeeding, kangaroo mother care, admission weight, and jaundice are significant predictors of survival. Therefore, considerable attention such as intensive photo-therapy, optimal calorie feeding, oxygenation, exogenous post-natal surfactant administrations and good thermal care should be given for admitted preterm neonates. Moreover, the study didn't investigate the true causality of some predictors like maternal socio-economic and nutritional status on the incidence of preterm mortality. Therefore, we recommend a multi-center prospective cohort study to show the public health importance of these and other predictors on preterm mortality.

## Data Availability Statement

The original contributions presented in the study are included in the article/supplementary material, further inquiries can be directed to the corresponding authors.

## Author Contributions

DB worked on conceiving and designing the study, training and supervising the data collectors, interpreting the result, and preparing the manuscript. The co-authors namely AW, WW, HH, and WB played their roles in analyzing and interpreting the result. Moreover, the co-authors wrote the manuscript. All authors were involved in reading and approving the final manuscript.

## Conflict of Interest

The authors declare that the research was conducted in the absence of any commercial or financial relationships that could be construed as a potential conflict of interest.

## Publisher's Note

All claims expressed in this article are solely those of the authors and do not necessarily represent those of their affiliated organizations, or those of the publisher, the editors and the reviewers. Any product that may be evaluated in this article, or claim that may be made by its manufacturer, is not guaranteed or endorsed by the publisher.

## Abbreviations

AHR: adjusted hazard ratio
ANC: antenatal care
AOR: adjusted odd ratio
ARR: adjusted relative risk
CI: confidence interval
CPAP: continuous positive airway pressure
CS: cesarean section
HMD: hyaline membrane disease
HR: hazard ratio
KMC: kangaroo mother care
LBW: low birth weights
NEC: necrotizing entro colitis
NICU: neonatal intensive care unit
OR: odd ratio
PIH: pregnancy induced hypertension
PNA: peri natal asphyxia
PROM: premature rupture of membrane
RDS: respiratory distress syndrome
RR: relative risk
SVD: spontaneous vaginal delivery
UNICEF: united nation international children's emergency fund
WHO: world health organization.
